# Emerging Issues Questioning the Current Treatment Strategies for Lumbar Disc Herniation

**DOI:** 10.3389/fsurg.2022.814531

**Published:** 2022-03-28

**Authors:** Zhong Y. Wan, Hua Shan, Tang F. Liu, Fang Song, Jun Zhang, Zhi H. Liu, Kun L. Ma, Hai Q. Wang

**Affiliations:** ^1^Department of Orthopedics, The Seventh Medical Center of General Hospital of People's Liberation Army (PLA), Beijing, China; ^2^Institute of Integrative Medicine, Shaanxi University of Chinese Medicine, Xi'an, China; ^3^Department of Stomatology, The Specialty Medical Center Rocket Force of People's Liberation Army (PLA), Beijing, China; ^4^Department of Orthopedics, Baoji Central Hospital, Baoji, China; ^5^Department of Cardiac Surgery, Xijing Hospital, Air Force Medical University, Xi'an, China; ^6^Department of Orthopedics, Yongchuan Hospital of Chongqing Medical University, Chongqing, China

**Keywords:** adjacent segment disease, instrumentation, lumbar disc herniation, lumbar fusion, metallosis

## Abstract

Lumbar disc herniation is among the common phenotypes of degenerative lumbar spine diseases, significantly affecting patients' quality of life. The practice pattern is diverse. Choosing conservative measures or surgical treatments is still controversial in some areas. For those who have failed conservative treatment, surgery with or without instrumentation is recommended, causing significant expenditures and frustrating complications, that should not be ignored. In the article, we performed a literature review and summarized the evidence by subheadings to unravel the cons of surgical intervention for lumbar disc herniation. There are tetrad critical issues about surgical treatment of lumbar disc herniation, i.e., favorable natural history, insufficient evidence in a recommendation of fusion surgery for patients, metallosis, and implant removal. Firstly, accumulating evidence reveals immune privilege and auto-immunity hallmarks of human lumbar discs within the closed niche. Progenitor cells within human discs further expand the capacity with the endogenous repair. Clinical watchful follow-up studies with repeated diagnostic imaging reveal spontaneous resolution for lumbar disc herniation, even calcified tissues. Secondly, emerging evidence indicates long-term complications of lumbar fusion, such as adjacent segment disease, pseudarthrosis, implant failure, and sagittal spinal imbalance, which get increasing attention. Thirdly, systemic and local reactions (metallosis) for metal instrumentation have been noted with long-term health concerns and toxicity. Fourthly, the indications and timing for spinal implant removal have not reached a consensus. Other challenging issues include postoperative lumbar stiffness. The review provided evidence from a negative perspective for surgeons and patients who attempt to choose surgical treatment. Collectively, the emerging underlying evidence questions the benefits of traditional surgery for patients with lumbar disc herniation. Therefore, the long-term effects of surgery should be closely observed. Surgical decisions should be made prudently for each patient.

## Introduction

As one of the most burdensome health issues globally, low back pain (LBP) causes vast expenditures in treatment and sick leave from work (1). According to the Global Burden of Disease Study 2013, LBP is one of the most common musculoskeletal diseases amongst 301 acute and chronic diseases and injuries based on data from 188 countries during 1990–2013 (2). Degenerative diseases of the intervertebral discs, such as lumbar disc herniation (LDH, MeSH: intervertebral disc displacement), represent part of the most common causes of LBP (3). The prevalence of LDH is 2% in the general population (4) and 1.42% in adolescents (5), according to SweSpine. Except for the presence of cauda equina syndrome, plegia, and sensory-motoric deficits, controversy still exists regarding the indications for surgical intervention.

Whether to choose conservative measures or surgical treatments is still controversial for LDH in some areas (6). The traditional surgical procedures of LDH are various according to the disease, including pure decompression, decompression with non-instrumented fusion, decompression with instrumented fusion, minimally invasive decompression with fusion, decompression associated with a dynamic stabilization system, etc. Patients with isolated herniated lumbar discs causing radiculopathy are recommended to undergo the primary disc excision operation, such as open discectomy, endoscopic discectomy, or laminectomy in the guideline. Lumbar spinal fusion is not recommended as a routine treatment for these patients. However, lumbar spinal fusion is recommended for patients with herniated discs who have severe degenerative changes or obvious intersegmental instability caused by the herniated discs. Besides, reoperative discectomy and fusion is a potential treatment option in patients with recurrent disc herniations associated with significant deformity, instability, or chronic axial low back pain (7). Reoperative discectomy and fusion are believed superior in minimizing mechanical instability and recurrence compared to reoperative discectomy for the recurrent cases (8).

A retrospective study consisting of 18,590 patients with LDH who underwent surgical treatment showed that open discectomy was the most common procedure (68.9%) in the primary operation, followed by endoscopic discectomy (16.1%), laminectomy (7.9%), fusion (3.9%), and nucleolysis (3.2%) (9). Although pure decompression is the most recommended surgical procedure for the purely herniated with neurological symptoms, the reoperation rates were considerably high, with 18.6, 13.8, and 12.4% after laminectomy, open discectomy, and endoscopi**c** discectomy, respectively (9). As a matter of fact, removing part of the lumbar disc might induce a secondary complex situation that can bring spinal instability (10). And it turns out an initial lumbar discectomy for the patients with LDH is statistically associated with an increased likelihood of lumbar fusion in the future (11).

The selection of treatment strategy for LDH should be based on the severity of the disease and the patient's overall condition (12). Whether to choose non-surgical or surgical treatment and which surgical procedure is selected depends on the severity of symptoms and the clinical-pathological correlate. However, the decision-making of treatment strategy is partially preference-sensitive, depending on the surgeon's preference, which is influenced by the doctors' experience and patients' expectations.

Although surgical treatment has been demonstrated as an effective treatment strategy with the advantages, such as rapid symptoms relief, increased stability, facilitated bone healing, and restored alignment, several disadvantages or complications in the long term have also been noted (13). These complications, i.e., adjacent segment degeneration, metallosis, and additional ionizing radiation exposure, have been widely reported in the last two decades. There is accumulating evidence that questions the benefits of traditional surgery for patients with LDH (14, 15). Given that the pros of surgical treatment have been well-documented in the literature, we will emphasize the cons from four aspects in the current review.

## Natural History Issue of LDH

### Immune Privilege of NP

#### Closed Niche

**Figure 1:**
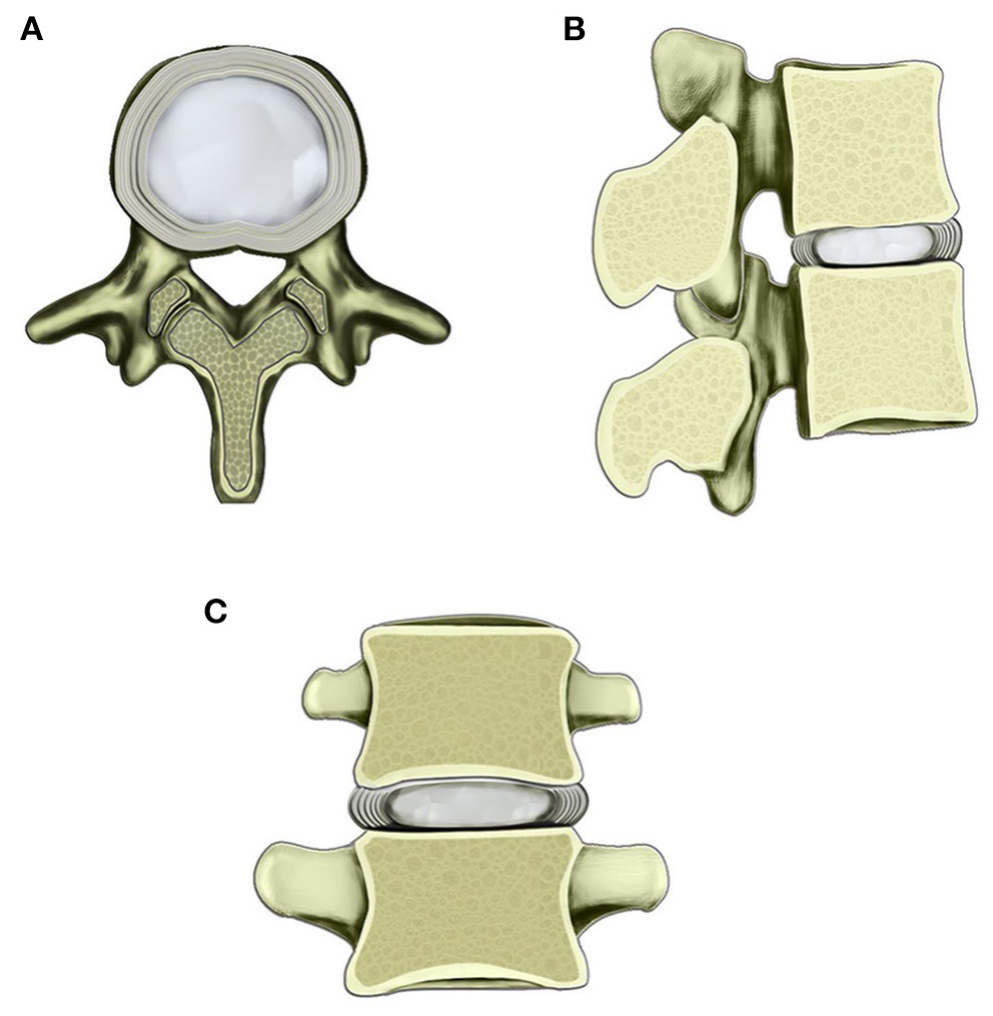
Schematic diagram of the normal lumbar disc within the scope of the vertebral body. **(A)** Transverse view of normal lumbar disc. **(B)** Sagittal view of lumbar disc and adjacent vertebral bodies. **(C)** Coronary view of lumbar disc and adjacent vertebral bodies. The border of the central nucleus pulposus and surrounding AF is clear.

Physiologically, the human lumbar disc comprises three subparts, i.e., the sandwiched central nucleus pulposus (NP), peripheral annulus fibrosus (AF), and adjacent cartilage endplates. The local environment of NP cells is similar to a closed niche (16) (Figure 1). Furthermore, the disc belongs to one of the largest avascular structures in the human body. The blood vessels and innervations terminate in the outer layer of AF of the healthy disc (17). The closest distance from the center of NP to the blood supply is as far as 7–8 mm (18). The nutrition of NP mainly derives from the osmosis of cartilage endplate and AF (18, 19). Therefore, human NP in the disc remains untouched from the immune system, being the physiologic basis of human discs as immune-privileged organs.

#### FasL-Fas Network as Underlying Mechanisms of NP Immune-Privilege

FasL (Fas ligand, CD178) localizes in human NP cells strategically as a death factor, which can bind with Fas (CD95, death receptor) of invasive immune cells and endothelial cells (20, 21). The binding of FasL to Fas induces apoptosis (22) of the invasive immune cells, maintaining the immune-privilege characteristic of the intact human NP (23). It has been reported that the cells' morphological alterations and chromosomal DNA degradation in apoptosis occur within a few hours *in vitro* (24).

#### Breakdown of the Immune Privilege and Disc Degeneration

Kaneyama et al. (25) found a significant decrease of FasL expression in the degenerated discs compared with the non-degenerate discs, implying the potential protective role of FasL against degeneration. Fas and FasL's expression on stabbed-disc cells is significantly higher than those in normal disc cells (26). When the physiological barrier is damaged, an autoimmune reaction is evoked. Immune cells expressing with FasL bind with NP cells expressing with Fas, which induces the NP cells apoptosis. At the same time, up-regulated FasL in NP co-expressing with Fas induces apoptosis of disc cells *via* the paracrine pathway. Deregulated FasL and Fas contributing to the abnormal apoptosis of NP cells may be possible pathogenesis of intervertebral disc degeneration (IDD).

Emerging evidence indicates that various physiologic and pathologic processes are regulated by the coding (mRNAs)-non-coding RNA (ncRNA) network. Types of ncRNAs, such as microRNA (miRNA) and long non-coding RNA (lncRNA), are involved in various physiologic and pathologic processes in IDD, which were reported previously. We found that several miRNAs are differentially expressed in degenerative NP, including the down-regulated miR-155. Further investigation revealed that miR-155 plays a regulatory role in FasL-Fas apoptotic signaling pathway. Deregulated miR-155 increases the expression level of Fas-associated death domain-containing protein (FADD) and caspase-3, promoting Fas-mediated apoptosis in IDD (27). Following that, **a** lncRNA-mRNA microarray analysis of human NP was conducted in 2014 (28). Up-regulated expression of enhancer-like lncRNA RP11-296A18.3 was observed, inducing the overexpression of Fas-associated protein factor-1 which induces the Fas-mediated apoptosis of NP cells at last. Subsequently, Cui et al. indicated that another lncRNA, MAGI2-AS3, is down-regulated in IDD, which is inversely related to the FasL level in NP cells (29). Decrease expression of lncRNA MAGI2-AS3 may promote FasL expression and trigger the FasL-Fas apoptotic signaling pathway, resulting in the apoptosis of NP cells. We addressed the Fas-FasL interacting network between NP, immune cells, and certain modulation factors (21), organizing global researchers for a hot topic issue on IDD (30).

#### Endogenous Repair Basis

During the regeneration process of various organs, endogenous repair exists, including liver, gut, skin, muscle, kidney, and bone (31). Each organ has a specific capacity for endogenous repair. Accumulating evidence indicates that endogenous repair exists in the human disc, with progenitor cells as crucial contributors (32, 33). In 2007, Risbud et al. (34) first identified human NP and AF cells expressing specific stem cell types of surface markers from degenerative discs. Moreover, these cells can differentiate into chondrogenic, osteogenic, and adipogenic lineages. After that, multiple lines from *in vivo* and *in vitro* studies indicated the existence of progenitor cells in human intervertebral discs. Intervertebral disc cells expressing Tie 2 represent a subtype progenitor cell group with discogenic differentiation potential and enhanced regeneration (35).

Besides these basic lines of evidence, various clinical factors contribute to the disruption of the barrier, including trauma/microtrauma during daily life, aging/pathologic alterations (such as scoliosis) with cartilage endplate (CEP) degeneration, iatrogenic, congenital factors, and/or vertebral endplate morphology (36).

### Emerging Etiology Evidence of LDH

#### Clinical Evidence of Spontaneous Resorption of Herniated Intervertebral Discs

Cribb et al. reported a dramatic regression of massive herniation in 14/15 patients after an average 24 months follow-up (range: 5–56 months) (37). Compared with bulges and focal protrusions, broad-based herniation and sequestrations improve more (38). Not only massive soft herniation but large calcified disc herniation could be absorbed as well (39). Other spinal herniation, such as cervical/thoracic disc herniation with/without calcification, has also been reported with spontaneous resolution (40, 41).

**Figure 2:**
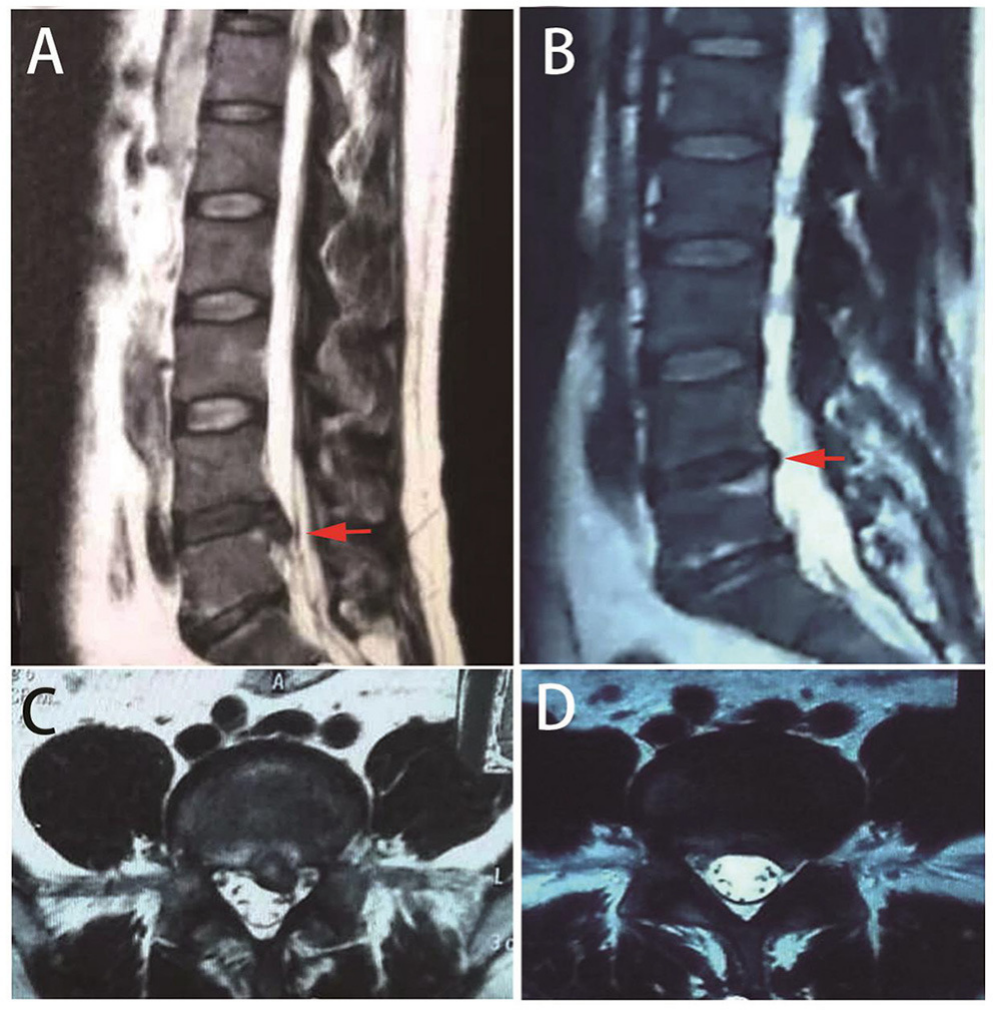
Repeated MRIs of a typical case with spontaneous resolution. A 39-year-old male patient presented with low back pain and sciatica. MRI indicates lumbar disc herniation at L5/S1 [**(A,C)** red arrows]. One year later, repeated MRI indicates herniation resolution [**(B,D)** red arrows].

Repeated MRIs revealed the shrinkage of herniated discs gradually, with 76% or more absorbed in 1 year (Figure 2). Moreover, Panagopoulos et al. summarized 12 studies in a systematic review (42). Amongst 901 middle-aged LDH patients, 15% to 93% were partially or entirely relieved by 1 year with repeated MRI observations. Zhong et al. conducted a meta-analysis with 11 cohort studies and revealed that the overall incidence of spontaneous regression in LDH patients was 66.66%, with a regional difference (43).

Clinical symptoms, such as sciatica and motor and sensory deficits, can gradually improve in non-surgical treatment LDH cases (44, 45). However, changes in the size of herniated intervertebral discs on MRI are not significantly correlated with the development of clinical symptoms. For instance, sciatica is influenced by multiple factors. Not only the relief of the mechanical compression but also the decreased severity of the inflammatory or chemical irritation contribute to the alleviation of the clinical symptoms (46).

#### Autoimmune Response and Inflammation Cascade Underlying the Spontaneous Resorption of Herniated Intervertebral Discs

**Figure 3:**
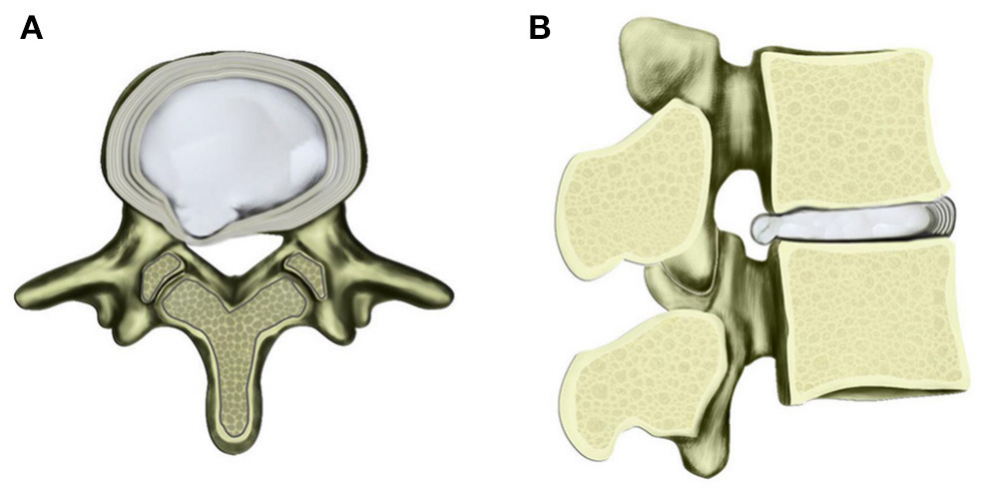
Schematic diagram of contained herniated lumbar disc with transverse **(A)** and sagittal views **(B)**. Under multiple factors, the nucleus pulposus protrudes toward posterior and lateral direction with AF fibers ruptured to a certain extent.

Human intervertebral discs, particularly NP, belong to immune-privileged sites. The initial immune-privileged scenarios change dramatically when NP protrudes out from the closed niche (Figure 3). The herniated tissue is recognized as a foreign antigen by the autoimmune system, attracting immune cells and auto-antibodies, triggering an autoimmune response, and inflammation cascade (47).

In 1965, Bobechko and Hirsh revealed that an autoimmune response is induced when NP of rabbits is exposed to the systemic circulation, giving rise to the auto-antibodies production in lymph nodes (48). Subsequently, a high level of IgG and IgM was found in herniated human intervertebral discs (49, 50). Satoh et al. indicated that the antigen-antibody complexes exist particularly in the pericellular space of NP cells rather than the NP cell membrane. This implied that the newly produced substances are surrounding the NP cell, such as polysaccharides, are playing roles of auto-antigen in the immune response (51). Later, evidence of several studies suggested that not only humoral immune response but cellular immune response also exists in the autoimmune response to the herniated substance. Geiss et al. reported that activated T and B cells infiltration is observed in autologous porcine NP exposed to the autoimmune system (52), including IL-4-producing Th2 cells, which participate in the humoral immune system response (53). By using the immunohistochemical analyses, Ikeda et al. (54) and Park et al. (55) found that a small number of T cells and many macrophages are infiltrating the herniated NP tissue. Murai et al. indicated that macrophages and NK cells are the early immune responder after the exposure, then are the T and B cells (56).

Neovascularization has been widely reported contributing to the resorption mechanism. With many newly formed vessels around the disc fragments, granulation tissue was observed on the herniated NP tissue (57). Several inflammatory factors or cytokines, such as tumor necrosis factor–α, midkine, vascular endothelial growth factor, and fibroblast growth factor 2, have been identified as the inducer of angiogenesis (45, 58–62). Macrophages migrate through the newly formed vessels and converge around the disc fragments (58, 63, 64). The infiltrating macrophages produce high levels of matrix metalloproteinase (MMP), including MMP-3 and MMP-7 (65). Cells from herniated discs undergo autoself-induced apoptosis progress *via* autocrine or paracrine Fas-FasL mechanisms (55). A high level of matrix enzyme degrades the aggrecan and collagen in the herniated material. Finally, the fragment of the tissue and apoptotic cells is absorbed by the macrophages and disc cells *via* phagocytosis (65–67).

#### The Hypothesis of the Spontaneous Regression in LDH Natural History

There are three hypotheses to explain the mechanism of spontaneous resorption in LDH. The first hypothesis is the dehydration and shrinkage of the herniated material (68). The second mechanism is supposed that the herniated disc, which is elastic and not separated from the main part in the intervertebral disc space, retracts back to the central place gradually (69). The third is the mechanism mentioned in the previous segment. The herniated disc is identified as a foreign antigen, inducing an autoimmune response and inflammatory cascade. Then, the matrix substance and apoptotic cells are degraded and absorbed by the macrophages *via* phagocytosis (52). It is supposed that all of the three mechanisms contribute to the spontaneous resorption process (70).

## Non-Surgical Treatment—the Foremost Option for LDH Patients Without Serious Symptoms

LDH is treated with surgical or non-surgical measures. Non-surgical treatments of LDH include various methods, such as bed rest, lumbar supports, physical therapy, spinal manipulation, oral analgesics, muscle relaxants, epidural steroid injections, and behavioral therapy (71). Except for the presence of cauda equina syndrome and neurologic impairment, controversy still exists regarding the indications for surgical intervention. North American Spine Society's (NASS) clinical guideline for LDH with radiculopathy indicated that the evidence in a recommendation for urgent surgery is insufficient for LDH patients with motor deficits (72). Several prospective controlled studies suggested that patients undergoing non-surgical treatment should only switch to surgical treatment with exacerbated symptoms (73, 74). Either surgical or conservative measures are suggested effective both in the short and long term for patients with less severe symptoms (72). The recommendation of Danish national clinical guidelines of recent onset lumbar nerve root compression advised at least 12 weeks of a conservative treatment to LDH patients before being considered for operation unless ongoing severe symptoms such as severe pain and disability (75). However, the North American Spine Society's (NASS) clinical guideline for LDH with radiculopathy suggested that LDH patients whose symptoms are severe enough to warrant surgery seek surgical intervention in 6 months. They indicated that earlier surgery (within 6 months to 1 year) is related to faster recovery and better long-term outcomes (72).

Although surgery is effective for LDH patients with radiculopathy in the short term, the surgical complication, repeat operation, and symptomatic recurrent LDH are unavoidable frustrating issues for part of them. A meta-analysis including 34,639 surgical cases of LDH revealed that the overall incidence of complications is 2.7%, while 2.1% of the patients had repeat operations within 3 months (76). Consistently, another study conducted in the US revealed that the average reoperation rate for LDH patients is 1.9% at 90 days, 6.4% at 1 year, and 13.8% at 4 years. Decompressions without fusion account for the majority of re-operative procedures (73%), while fusion with or without decompression nearly makes up for the rest (25.7%) (77). Apart from the undesirable operational effect, symptomatic recurrent LDH is another cause of reoperation. A small part of patients (5–15%) experience unfavorable events, and 4% to 6% undergo surgery in 2 years (78–81).

Although the surgical intervention has the advantages, i.e., rapid relief of symptoms and faster recovery of neurological deficits in the short term (13), several randomized controlled trials (RCTs) showed that the difference between conservative and surgical treatment in LDH patients with radiculopathy is non-significant 1 year later after diagnosis (82). Considering that spontaneous resorption of herniated discs commonly exists in the natural history of LDH, symptoms in a proportion of the patients will resolve on their own. Parts of the patients with LDH, particularly those without serious symptoms, are likely to benefit from the conservative treatment. Part of the patients' clinical symptoms will be alleviated or even completely disappear in a short time (83). Therefore, we suggested that non-surgical treatment is the foremost recommended measure for LDH patients without serious symptoms, such as cauda equina syndrome and motor deficits, which may achieve the same clinical outcomes and avoid various discomfort caused by the operation.

### A Long-Term Complication of Lumbar Fusion

In the US, the annual incidence of spinal fusion surgeries has increased over 600% from the 1990s to 2011. Nowadays, 450 000 spinal fusion cases are performed yearly (84). The national trend has been persistent during different observational periods (85). Whereas, spinal fusion with instrumentation increases healthcare expenditures, a surge of serious complications associated with the fusion has been observed as well. Increased local stress and compensatory motion on the non-operated adjacent levels after fusion procedure were both reported, giving rise to many problems, such as adjacent segment diseases (ASD) (86).

### Adjacent Segment Disease

ASD was defined as presenting a new clinical symptomatic degenerative disease corresponding to an adjacent level following spinal fusion at an index segment (87). ASD was represented by a series of pathological changes at the adjacent segments, such as disc height loss, disc herniation, canal stenosis, osteophyte formation, spondylolisthesis, and scoliosis (88). The incidence of ASD has been reported to vary from 4 to 45.7% of patients undergoing mono-segmental and multi-level fusion (88–91). Strikingly, multiple-repeated ASD following posterior lumbar interbody fusion of a single segment has been reported (92). Four patients among 1,112 consecutive patients developed multiple-repeated ASD with multiple repeated surgeries, even fusion upper to T1.

The etiology, incidence rate, and treatment strategies for ASD remain undefined. Risk factors of ASD include obesity, natural degeneration with aging, increased stress in intra-disc, preoperative disc degeneration, intraoperative superior facet joint violation, fusion at more than four levels, adjacent cranial segment, the upper shift of lumbar motion center, and decreased sacral slope (87, 88, 93–95). The incidence rate of ASD varies in terms of studied patient samples, follow-up time frame, the number of fusion segments, fusion techniques (180 or 360 degrees), and patients' age (87, 94). Regarding treatment strategies, a systematic review and meta-analysis indicated little available evidence addressing the optimal treatment options for patients with ASD for stenosis with or without instability (96).

Adjacent segment degeneration in the radiograph is the initial stage of ASD, referring to the degeneration of adjacent levels in diagnostic imaging (such as MRI) without clinical symptoms. Several researchers investigated the prevalence of radiographic adjacent segment degeneration and reported that the incidence is ranged from 9 to 27% in the lumbar spine (97, 98). A considerable proportion of the patients underwent an additional operation in the next few years. A series of risk factors were revealed in the published paper for the adjacent segment degeneration, which is similar to ASD (99). A systematic review indicated that the difference among the fusion procedures results in the variation of incidence in adjacent segment degeneration (99). Both aspects have been suggested as the key factors to avoid adjacent segment degeneration, including the reservation of posterior elements in the fusion procedure and perioperative treatment of osteoporosis.

### Other Complications Associated With Fusion Surgery

A systematic review of the literature of lumbar fusion for degenerative disorders, including 160 studies, revealed that the overall complication rate of lumbar fusion procedure is 14% (100). Apart from ASD, other long-term complications associated with fusion surgery, such as pseudarthrosis, implant failure, and sagittal spinal imbalance, were also widely reported in the literature (101, 102). The overall fusion rate for patients undergoing lumbar fusion procedures was reported as 88.5% (100). Smoking, metabolic disorders, surgical instrumentation and technique, and fusion location have been demonstrated as the risk factors for pseudarthrosis (103, 104). In addition to this, osteopenia and osteoporosis have been suggested as another risk factor for pseudarthrosis, and implant failure, such as screw loosening (105, 106). Post-operative back pain was reported in the patients undergoing lumbar fusion procedures. In-depth investigation showed that poor post-operative spinal sagittal alignment is related to prolonged back pain (107). Apart from that, the sagittal spinal imbalance was also associated with the body imbalance, which induces falls (108). The causes of sagittal imbalance are multifactorial, including pseudarthrosis at the lumbosacral junction, adjacent segment disease, and high pelvic incidence (109). The restoration or correction of sagittal alignment is important to the patients' surgical outcome and quality of life.

## Metallosis Issue

### Metal Debris and Elevated ion Level in Arthroplasty

Due to electrochemical corrosion and/or mechanical wear, surgical metallic implants have gained increasing attention in recent years. As early as 1973, Coleman et al. (110) presented the first line of evidence on a raised level of cobalt and chromium in the blood and urine of patients with metallic total hip replacements. Submicrometer metal particles within macrophages in the liver and/or the spleen were observed in patients undergoing primary and revision total hip arthroplasty (111).

The elevated level of systemic metal particles accumulating in the end organs, such as the heart (112), liver (111), and spleen (111), resulting in systemic metal toxicity, such as cobalt toxicity (113–116), even causing death (117). Apart from that, intracellular phagocytosis of particulate debris by macrophages can trigger the release of proinflammatory cytokines in the surrounding tissue, inducing aseptic fibrosis, local necrosis, or loosening of a device secondary to metal corrosion (118). Such type of metal debris staining complication is termed metallosis (119). Metallosis is a potentially fatal complication originally found in patients after arthroplasty, which is generally associated with metal or non-metallic implant wear (120). By analyzing whole blood metal and ion levels in 185 patients undergoing bilateral Birmingham Hip Resurfacing, Matharu et al. (121) proposed that the optimal threshold was 5.5 μg/L for distinguishing patients with and without adverse reactions metal debris.

### Systemic and Local Reactions Related to Spinal Metallic Implants

Joint prostheses and spinal instrumentation have different biomechanical effects on the human body. Regardless of corrosion mechanisms, mechanical wear is the predominating reason for the metallosis after arthroplasty, whereas fretting wear is the primary cause for metallosis after spinal instrumentation (119). It is generally speculated that the inevitable micromotion at the metal-metal junctions may lead to fretting corrosion and production of the particulate metallic debris after spinal instrumentation. Spinal metallic implants are currently made of titanium alloy, containing 90% titanium, 6% aluminum, and 4% vanadium. Other metal components exist in spinal implants containing niobium. It is widely reported that Ti6Al4V is highly susceptible to fretting corrosion due to a mixed microstructure when the titania passivation layer is disrupted (122). In contrast to these findings, a long-term test showed that the titanium and cobalt chrome constructs are more resistant to fretting corrosion than stainless steel (123).

In 1999, Wang et al. (124) reported that wear debris is generated in the tissue surrounding titanium spinal implants from nine patients undergoing prior lumbar decompression and fusion procedure and reoperation. Metal levels were higher in patients with pseudarthrosis than patients with a solid spinal fusion (30.36 μg/g of dry tissue vs. 0.586 μg/g of dry tissue). In 2003, Kaisai et al. (125) studied metal concentrations in the serum and hair of 46 patients with titanium alloy spinal implants, using inductively coupled plasma emission spectroscopy. Accordingly, they noted that one-third of involved patients exhibited higher serum or hair metal concentrations following surgery. Titanium or aluminum may have distant organ accumulation from the spinal implants. In 2008, Richardson et al. (126) reported higher serum titanium levels in 30 patients with titanium alloy spinal instrumentation prospectively in comparison with controls (2.6 vs. 0.71 μg/L), using high resolution inductively coupled plasma-mass spectrometry [HR-ICP-MS, detection limit for titanium as 0.25 μg/L (ppm)]. Instrumented spinal fusion can result in abnormally elevated serum titanium, aluminum, and niobium levels in pediatric patients undergoing instrumented spinal arthrodesis to correct scoliosis and kyphosis (127–129). A systematic review concerning the concentration of metal ions following multi-level spinal fusion, which includes 18 studies and encompasses 653 patients, showed that metal ions are elevated after instrumented spinal fusion, notably Cr levels from stainless steel implants, and Ti from titanium implants (130). Moreover, serum metal ion levels correlate positively with fusion segments and numbers of spinal implants.

### The Harmful Effect of Metallosis After Spinal Implantation

Accumulating evidence has unraveled local and systemic reactions to metal spinal implants. Metal particulate debris deposited in the soft tissue surrounding spinal implants was shown to activate a macrophage response that triggers the release of proinflammatory cytokines, leading to mild chronic inflammation, and stimulating the formation of the metal debris granuloma (131). The chronic inflammation irritated by the metal debris was suggested to be associated with the late operative site pain, which is eliminated until the implant is removed (132). Several researchers reported that the intraspinal extradural granuloma resulting from the foreign body reaction to the metallic wear debris contributes to the compression of the neurological elements and neurological symptoms mimicking the lumbar spinal stenosis (133–135). Moreover, metal debris has been shown to induce the mature osteoclast precursor and apoptosis of osteoblast, increase the peri-prosthetic bone resorption, and inhibit osteogenesis. These effects result in implant debris-related osteolysis, aseptic fibrosis, local necrosis, or implant loosening (136–140).

Metal debris has also been displayed to stimulate the immune system to induce a series of type IV delayed-type IV hypersensitivity responses (141, 142). These immunogenic reactions are presented as anorexia, fatigue (143), severe dermatitis (144), urticarial (145), and vasculitis (145). In addition, much evidence indicates that degraded metal particles from spinal metallic implants can enter the systemic blood circulation and deposit in the heart, liver, and spleen. The average level of serum titanium is similar to that of patients undergoing arthroplasty. Although a few findings have been reported, the long-term impact of elevated serum metal concentrations on patients with a spinal implant is not entirely clear. Furthermore, there has been no established threshold above which metal concentrations will be toxic after the spinal instrumented surgery. Removing the spinal implantation at the right time may be a method to avoid metallosis.

## Spinal Implant Removal

The latest updated guidelines (NG59) drafted by the National Institute for Health and Care Excellence in the UK (https://www.nice.org.uk/guidance/ng59) states that fusion for non-specific low back pain should be strictly used only for RCTs (146). The guidelines reflect those lessons obtained from clinical practice and reports. There have been no established indications for spinal implant removal until now. Therefore, whether to proceed depends on the surgeon's preference.

Even though the application of spinal instrumentation increased the probability of successful spinal fusion, stress shielding induced osteoporosis on account of the rigid fixation and increased the risk of recurrent fracture after implant removal (106, 147, 148). Acquired spondylolysis has been a well-recognized stress fracture after posterior lumbar fusion since 1963 (149). Nevertheless, adverse events have been reported following pedicle screw constructs removal, including pedicle stress fracture due to iatrogenic weakness of the pedicles following removal (150, 151), vertebral compression fracture within a solid lumbar fusion mass (152), or recurrent vertebral fracture following pedicle screw removal for index burst fracture (153). Therefore, the surgeon should attach attention to the implant removal time and method, avoiding implant removal failure.

## Other Challenges

Besides those mentioned above existing challenging issues, there are other questions to be solved, including decreased quality of life due to lumbar rigidity radiation exposure from perioperative and follow-up diagnostic imaging. In addition to common clinical outcome measures for lumbar spine surgery, indicators have been noted reflecting lumbar rigidity due to the decrease in kinematic units following lumbar fusion. Sciubba et al. (154) evaluated the impact of stiffness on activities of daily living following instrumented total lumbar fusion. The most affected activities of daily living included dressing or bathing the lower half of the body and performing personal hygiene functions after toileting.

By adding instrumentation, patients have to experience additional ionizing radiation exposure for the orientation of pedicle screws during surgery with fluoroscopy (155–157) observations on repeated radiographs for clinical outcome follow-up. Compared to pure decompression, adding instrumentation will result in more cumulative radiation exposure for surgeons, medical staff in operating theaters, and patients. Importantly, the awareness of such potential harms is low amongst medical professionals (158) and patients (159, 160).

Residual and recurring back pain after surgery is common in LDH surgeries. The proportion of patients reporting short-term (6–24 months) and long-term (>24 months) recurrent back pain ranged from 3–34% to 5–36%, respectively in a systematic literature review (79). Some people who have persistent pain postoperative are still unclear (161). Severe endplate changes, such as endplate avulsion, damaged the lumbar stability and maybe resulted in a higher recurrence rate and residual back pain (162, 163). LBP has been suggested to be associated with postural and structural asymmetries. CEP degeneration accompanied by loss of cellularity results in the asymmetric loading of the lumbar spine in LDH. Fusion surgery provides the stabilization and maybe correct asymmetry of the lumbar spine in part (164). However, the current operation aims not to solve the imbalance of load, nor can it completely solve the problem. Asymmetry of lumbar loading may still be one of the causes of residual back pain postoperative. The predictors of residual LBP after decompression included more severe LBP at baseline, degenerative scoliosis, and Cobb angle size (165).

As the second most mobile part of the human axis, the lumbar spine and related LDH have been a hot topic for the medical community (164). Nevertheless, LBP, most commonly caused by a herniated disc, is a constant concern (166). For decades, lumbar discectomy has been done by neurosurgeons as the general surgical practice to solve the disease (167, 168). The discovery of X-Ray brought about a shift of paradigm in the practice of neurosurgery (169). The introduction of microsurgical techniques led to an essential evolution in lumbar disc surgery (170). For those who have failed conservative treatment, surgery is the only option that must be considered. However, while the operation solves the symptoms, it also brings problems that can not be ignored. This paper summarizes the cons of surgical treatment from different perspectives. These summaries are useful supplements to the present literature, providing a unique vision for surgeons and patients who attempt to choose surgical treatment.

## Conclusions

**Figure 4:**
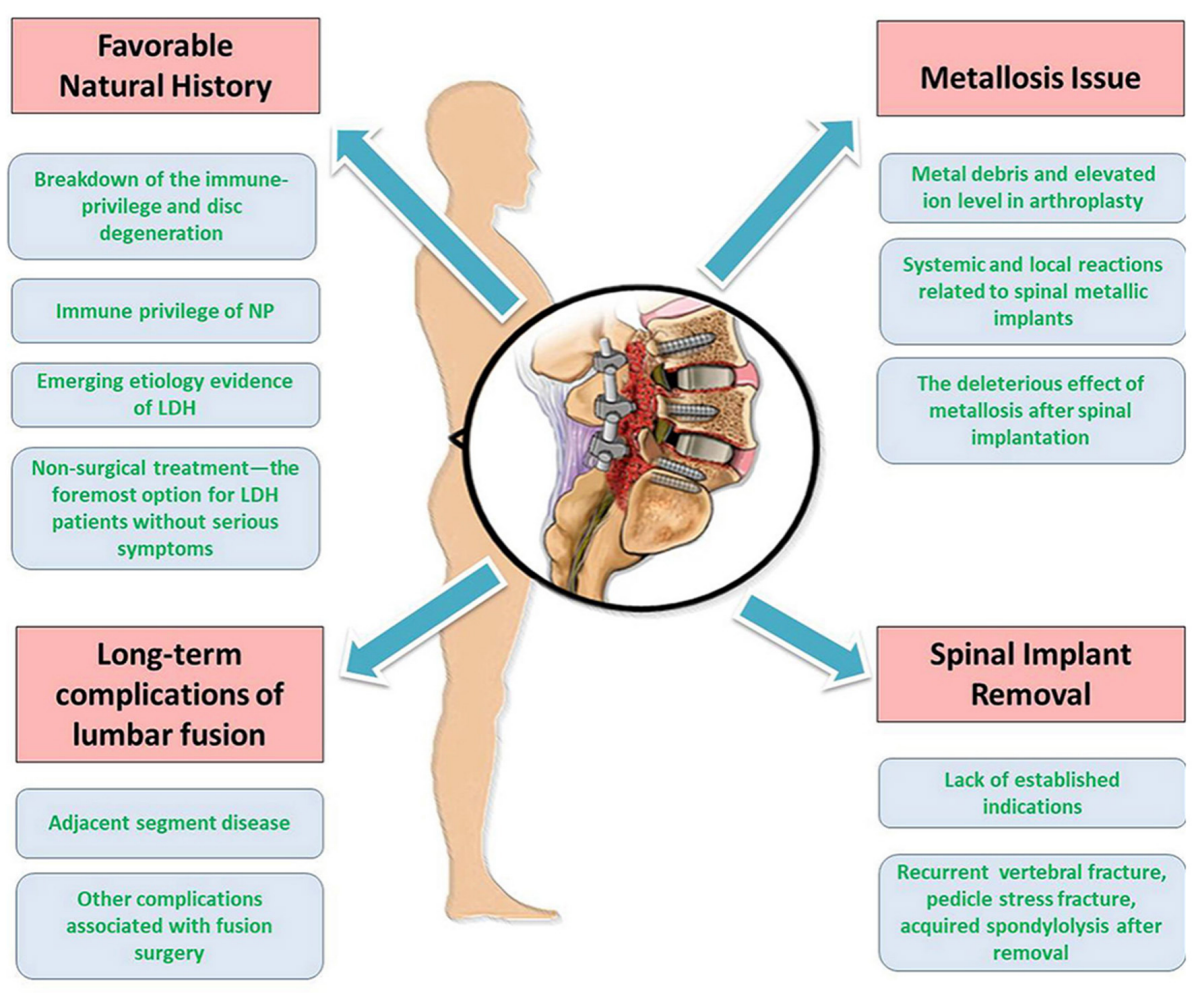
Summarizations of the different aspects of the review. There are tetrad critical issues pertaining to surgical treatment of LDH, i.e., favorable natural history, long-term complications of lumbar fusion, metallosis issue, and spinal implant removal.

Due to various triggering factors, lumbar surgeries with or without implementation increase rapidly with great health expenditures. In the review, we analyzed the tetrad critical issues about surgical intervention for LDH, i.e., favorable natural history, long-term complication of lumbar fusion, metallosis, and implant removal (Figure 4). Based on the limited evidence available so far, lumbar surgery solves the symptoms for the patients with LDH and brings a new series of unexpected problems. Therefore, the long-term effects of surgery should be closely observed. Surgical decisions should be made prudently for each patient.

## Author Contributions

HW conceived the study. ZW, HS, and TL investigated and retrieved the published papers, as well as wrote the original draft. FS, JZ, ZL, and KM reviewed and edited the final version of the manuscript. All authors have read and agreed to the final version of the manuscript.

## Funding

This work was supported by the National Natural Science Foundation of China (Grant no. 81572182).

## Conflict of Interest

The authors declare that the research was conducted in the absence of any commercial or financial relationships that could be construed as a potential conflict of interest. The reviewer F-LW declared a shared affiliation with the author ZL to the handling editor at the time of the review.

## Publisher's Note

All claims expressed in this article are solely those of the authors and do not necessarily represent those of their affiliated organizations, or those of the publisher, the editors and the reviewers. Any product that may be evaluated in this article, or claim that may be made by its manufacturer, is not guaranteed or endorsed by the publisher.

## Abbreviations

NP: nucleus pulposus
AF: annulus fibrosus
ASD: adjacent segment disease
CEP: cartilage endplate
FADD: Fas-associated death domain containing protein
FasL: Fas ligand
IDD: intervertebral disc degeneration
LBP: low back pain
LDH: lumbar disc herniation
lncRNA: long noncoding RNA
miRNA: microRNA
MMP: metalloproteinase
MRI: magnetic resonance imaging
NASS: North American Spine Society
ncRNA: noncoding RNA
RCTs: randomized controlled trials.
